# Persulfidome of Sweet Pepper Fruits during Ripening: The Case Study of Leucine Aminopeptidase That Is Positively Modulated by H_2_S

**DOI:** 10.3390/antiox13060719

**Published:** 2024-06-13

**Authors:** María A. Muñoz-Vargas, Salvador González-Gordo, Angeles Aroca, Luis C. Romero, Cecilia Gotor, José M. Palma, Francisco J. Corpas

**Affiliations:** 1 Group of Antioxidants, Free Radicals and Nitric Oxide in Biotechnology, Food and Agriculture, Department of Stress, Development and Signaling in Plants, Estación Experimental del Zaidín Spanish National Research Council, CSIC, C/Profesor Albareda 1, 18008 Granada, Spain; mangeles.munoz@eez.csic.es (M.A.M.-V.); salvador.gonzalez@eez.csic.es (S.G.-G.); josemanuel.palma@eez.csic.es (J.M.P.); 2 Instituto de Bioquímica Vegetal y Fotosíntesis, Consejo Superior de Investigaciones Científicas and Universidad de Sevilla, Avenida Américo Vespucio 49, 41092 Sevilla, Spain; aaroca@us.es (A.A.); lromero@ibvf.csic.es (L.C.R.); gotor@ibvf.csic.es (C.G.)

**Keywords:** aminopeptidase, fruit ripening, glutathione, hydrogen sulfide, nitric oxide, pepper

## Abstract

Protein persulfidation is a thiol-based oxidative posttranslational modification (oxiPTM) that involves the modification of susceptible cysteine thiol groups present in peptides and proteins through hydrogen sulfide (H_2_S), thus affecting their function. Using sweet pepper (*Capsicum annuum* L.) fruits as a model material at different stages of ripening (immature green and ripe red), endogenous persulfidated proteins (persulfidome) were labeled using the dimedone switch method and identified using liquid chromatography and mass spectrometry analysis (LC-MS/MS). A total of 891 persulfidated proteins were found in pepper fruits, either immature green or ripe red. Among these, 370 proteins were exclusively present in green pepper, 237 proteins were exclusively present in red pepper, and 284 proteins were shared between both stages of ripening. A comparative analysis of the pepper persulfidome with that described in Arabidopsis leaves allowed the identification of 25% of common proteins. Among these proteins, glutathione reductase (GR) and leucine aminopeptidase (LAP) were selected to evaluate the effect of persulfidation using an in vitro approach. GR activity was unaffected, whereas LAP activity increased by 3-fold after persulfidation. Furthermore, this effect was reverted through treatment with dithiothreitol (DTT). To our knowledge, this is the first persulfidome described in fruits, which opens new avenues to study H_2_S metabolism. Additionally, the results obtained lead us to hypothesize that LAP could be involved in glutathione (GSH) recycling in pepper fruits.

## 1. Introduction

Hydrogen sulfide (H_2_S) is a gasotransmitter generated in different subcellular compartments of plant and animal cells which has signaling functions under physiological and adverse environmental conditions [1,2,3,4,5,6,7,8,9]. From a chemical point of view, H_2_S is a weak acid that in an aqueous solution can be dissociated into hydrosulfide (HS^−^) and sulfide (S^2−^) anions. It is estimated that under physiological pH conditions, around 20% of H_2_S occurs in a non-dissociated form, and the rest is dissociated into HS^−^ and H^+^, whereas the amount of S^2−^ is very low at physiological pH [10]. H_2_S exerts its regulatory function throughout the generation of persulfides (RSSH) which involves their interaction with either oxidized thiol derivatives such as disulfides (RSSR′) and sulfenic acid (RSOH) or oxidized sulfur derivatives such as polysulfides (HSnS^−^ *n* ≥ 1) [11,12,13,14,15]. Thus, polysulfides can also react with glutathione disulfide (GSSG) to generate glutathione persulfide (GSSH) [16]. In the cases where thiol groups of susceptible cysteine residues are present in proteins, the corresponding persulfidated proteins will be generated [17,18]. As it happens with other thiol-based oxidative posttranslational modifications (oxiPTMs) such as *S*-glutathionylation, *S*-nitrosation, or S-acylation, the effect of persulfidation on protein function can be positive or negative.

Pepper (*Capsicum annuum* L.) is framed within the Solanaceae family and its fruits have great agroeconomic relevance worldwide since they are broadly consumed either fresh or processed [19]. For example, dry pepper powder is one of the most used spices in the preparation of cooking dishes for its aroma and flavor, but also in the preparation of sausages due to its antioxidant capacity which provides a high preservative capacity, and also for its taste [20]. At a nutritional level, pepper fruits are characterized to contain different compounds with antioxidant properties such as, for example, vitamins C, provitamin A, and E, flavonoids, phenols, or capsaicinoids for the spicy members of the genus [21,22,23,24,25].

In previous studies, we have provided evidence of the relevance of the metabolism of reactive oxygen and nitrogen species (ROS and RNS, respectively) in the ripening of sweet pepper fruits and how the application of exogenous nitric oxide (NO) could modulate these processes at different levels [26]. Thus, enzymes like peroxidases, superoxide dismutases (SODs), NADPH oxidases, and ascorbate peroxidases (APXs), among others, have been shown to undergo numerous changes at the transcriptomic, proteomic, and biochemical levels [27,28,29,30,31].

To our knowledge, the proteomic approach to identify persulfidated proteins (persulfidome) in higher plants has been carried out mainly in *Arabidopsis thaliana* organs (roots and leaves) under physiological and stress conditions [8,32,33,34,35,36] and common bean nodules at different developmental stages [37], but little information is available in edible fruits. This study provides the first comparative persulfidome of sweet pepper fruit at two stages of ripening including immature green and ripe red. Among the identified target proteins for persulfidation, the leucine aminopeptidase (LAP), which was previously shown to be modulated by NO [31], was selected to evaluate how H_2_S can affect its activity. Thus, the obtained data indicate that H_2_S exerts a positive effect on LAP activity which was also reversed by a reductant reagent (dithiothreitol), indicating that this process is reversible.

## 2. Materials and Methods

### 2.1. Plant Material

California-type sweet pepper (*Capsicum annuum* L., cv. Melchor) fruits were harvested between January and February (2021) from plants grown in plastic-covered greenhouses (Syngenta Seeds, Ltd., El Ejido, Almería, Spain). Fruits were selected and harvested without any external apparent injury at two developmental stages: green immature (G) and red ripe (R).

### 2.2. Dimedone Switch Method and Proteomics

Endogenous persulfidated proteins were labelled using the dimedone switch method previously described [38] with some modifications, and were identified using mass spectrometry analysis. Briefly, green and red pepper fruit samples were ground to a fine powder in liquid nitrogen, resuspended in cold lysis buffer (PBS, pH 7.4, 2% (*w*/*v*) SDS, and 1 mM EDTA), together with 5 mM 4-chloro-7-nitrobenzofurazan (Cl-NBF, Sigma-Aldrich, Madrid, Spain) and 1% (*v*/*v*) protease inhibitor, incubated at 37 °C for 30 min, and protected from the light. Then, a methanol/chloroform precipitation was performed, and protein-enriched pellets were washed with cold methanol and dried afterward. The dried proteins were dissolved into 50 mM PBS with 2% (*w*/*v*) SDS, incubated with 200 µM DCP-Bio1 (a biotinylated form of dimedone) (Kerafast, Inc., Vizcaya, Spain) at 37 °C for 1 h, precipitated, and finally dissolved in 1 × PBS with 2% (*w*/*v*) SDS and 1% protease inhibitor. Proteins were incubated with Sera-Mag Magnetic Streptavidin beads (Cytiva S.L., Hospitalet de Llobregat, Spain) at 4 °C overnight with agitation. The microtubes containing the magnetic beads were placed in front of a magnet and the beads were separated from the supernatant. Then, they were washed with PBS supplemented with 0.001% (*v*/*v*) Tween-20 only the first time and after with PBS 1× several times. After washing, the beads were recovered again and incubated with 2.25 M ammonium hydroxide overnight at 4 °C with agitation. The final supernatants containing the persulfidated proteins labelled with DCP-Bio1 were transferred to new microtubes, and the beads were discarded.

For mass spectrometry analysis, samples were digested with trypsin following a standard protocol by using reduction conditions with dithiothreitol (DTT) and the carbamidomethylation of the reduced cysteines with iodoacetamide. In total, 1 µg of proteins from both green and red pepper fruits was analyzed using liquid chromatography–mass spectrometry, using a gradient of 120 min in length. A 75 µm × 50 cm C18 column was used, at a flow rate of 250 nL/min, for the chromatography, and the analysis was carried out in a Thermo Orbitrap Exploris OE240 mass spectrometer working in DDA mode. The MS1 and MS2 spectra were used to launch searches against a *Capsicum annuum* L. database, downloaded from UniProtKB, consisting of 38,701 entries. Peaks v7 was used as the search engine, and only proteins identified with at least one unique peptide were shown, using the 1% value for FDR (False Discovery Rate) as a criterion. The following modifications (variables) of the peptides including oxidation were selected as possible (M): 15.99 Da; Carbamidomethylation: 57.02 Da; NBF: 163.00 Da (in Lys, Arg, and Cys); hydrolyzed DCP-Bio1: 168.08 Da (in Cys); and DCP-Bio1: 394.16 Da (in Cys).

### 2.3. Functional Analysis

The sets of proteins identified as potential persulfidation targets were used to perform different functional analyses. First, a functional enrichment analysis was performed in GO (Gene Ontology) terms, using the web tool g:Profiler (v2023) [36]. Then, the Python package GO-Figure! (v3) [37] was used to generate a more summarized and comprehensive version of the results.

Furthermore, the Mercator4 online tool (v6.0) [38,39] was used to assign functional categories to the bell pepper proteome (UniProt Proteome ID: UP000222542; accessed on 20 March 2024). The generated data were introduced into the Mapman software (v3.6.0RC1) [40,41] to visualize the metabolic pathways in which the persulfidated proteins were involved.

To compare the persulfidated proteins identified in pepper fruits and the persulfidome previously described in *Arabidopsis thaliana* [17], we used the BLASTP tool (v2.15.0) [42] using the Arabidopsis proteome (UniProt Proteome ID: UP000006548; accessed on 20 March 2024) as a database.

### 2.4. Fruit Extracts and Leucine Aminopeptidase (LAP) and Glutathione Reductase (GR) Activities

Fruit samples stored at −80 °C were homogenized in liquid nitrogen using an IKA A11 Extraction Mill. The pulverized plant material was weighed and 50 mM Tris-HCl buffer, pH 7.5, containing 0.1 mM EDTA, 1 mM MgCl_2_, 10% (*v*/*v*) glycerol, and 0.1% (*v*/*v*) Triton X-100, was added. The ratio of plant material to used buffer was 1:1.

For the spectrophotometric leucine aminopeptidase (LAP; EC 3.4.11.1) activity assay, L-leucine-*p*-nitroanilide (Leu-*p*-NA) was used as a substrate which, by the action of the LAP activity, generated L-Leu plus *p*-nitroaniline whose absorbance was measured at 410 nm. Briefly, the reaction mixture contained 50 mM K-phosphate buffer pH 7.5, 10 mM β-mercaptoethanol, and 1 mM Leu-*p*-NA plus fruit samples, and was incubated at 39 °C for 30 min. The reaction was stopped with 30% (*v*/*v*) acetic acid, centrifuged at 10,000× *g* for 10 min, and then the supernatant was measured a 410 nm [43]. One unit of LAP activity is defined as the change in one unit of absorbance at 39 °C which corresponds to the production of 1 nmol of *p*-nitroaniline per min, using an extinction coefficient ε_410_ of 10^4^ M^−1^cm^−1^ [44]. For comparisons of the LAP activity among treatments, Student’s *t*-test was used with a statistical significance of 5% (*p* ≤ 0.05).

Glutathione reductase (GR; EC 1.6.4.2) activity was assayed by monitoring at 340 nm the NADPH oxidation coupled to the reduction of oxidized glutathione [45].

Protein concentration in fruit extracts was determined using the Bio-Rad protein assay with bovine serum albumin as standard.

### 2.5. In Vitro Treatment of Pepper Samples

To investigate the potential modulation of the LAP activity in pepper fruits, two different in vitro assays were performed. (1) Samples from green pepper fruits were pre-incubated at 25 °C for 1 h at 25 °C and darkness with different concentrations of sodium hydrosulfide (NaHS), an H_2_S donor. Afterward, samples were further incubated for 1 h either in the absence or the presence of 50 mM dithiothreitol (DTT), as reductant agent. (2) Samples were pre-incubated with either 5 mM NaHS or 5 mM *S*-nitrosoglutathione (GSNO), a nitric oxide (NO) donor. Then, further incubations for 1 h were carried out in the presence of 5 mM GSNO or 5 mM NaHS, respectively. On the other hand, in the analysis of the simultaneous effect of H_2_S and NO, the samples were incubated for 30 min at 25 °C with the first donor, and then the second donor was added to complete together the full hour of incubation. In all cases, the solutions were freshly prepared before use. Then, either the GR or LAP activity was determined as described above.

## 3. Results

**Figure 1:**
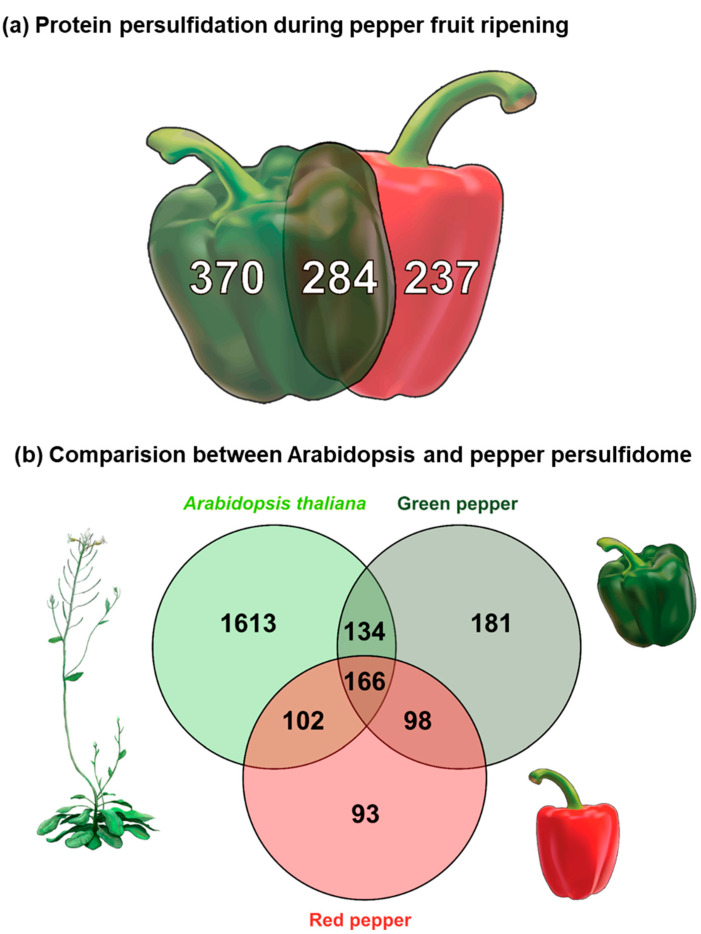
(**a**) Pepper persulfidome at different ripening stages, immature green and ripe red. A total of 891 proteins were identified, of which 370 proteins were exclusively present in green pepper, 237 were exclusively present in red pepper, and 284 were detected at both ripening stages. (**b**) Venn diagram of the comparative analysis of the persulfidomes from *Arabidopsis thaliana* leaves under physiological conditions and both green and red pepper fruits. In this last analysis, 2015 proteins from Arabidopsis and 814 from pepper fruits were considered.

Three biological replicates were analyzed for each ripening stage (unripe green and ripe red). Considering the biological replicates independently, we identified a total of 2143 unique proteins that could undergo persulfidation during sweet pepper fruit ripening. However, for subsequent analyses, we considered common persulfidated proteins in the replicates for each ripening stage. Accordingly, the number of endogenously persulfidated proteins identified in sweet pepper fruits was 891. Thus, out of them, 370 were exclusively present in green pepper and 237 were exclusively present in red pepper, whereas 284 proteins were common to both ripening stages (Figure 1a). Supplementary Tables S1 and S2 shows the list of all the proteins identified as likely to be endogenously persulfidated in immature green and ripe red pepper fruits.

A comparative analysis of the pepper persulfidome against that described for Arabidopsis leaves under physiological conditions [17] was achieved with the help of the BLASTP tool. This allowed us to find that 25% of Arabidopsis shared proteins with pepper fruits, although the restriction limits, established by the BlastP engine during the comparative analysis, could only evaluate 774 out of the 891 proteins identified in pepper fruits due to the redundancy in the identifiers between both species. Thus, comparing the persulfidated proteins reported in Arabidopsis leaves with those found in pepper fruits, 402 were shared proteins (Figure 1b) (see Supplementary Table S3).

**Figure 2:**
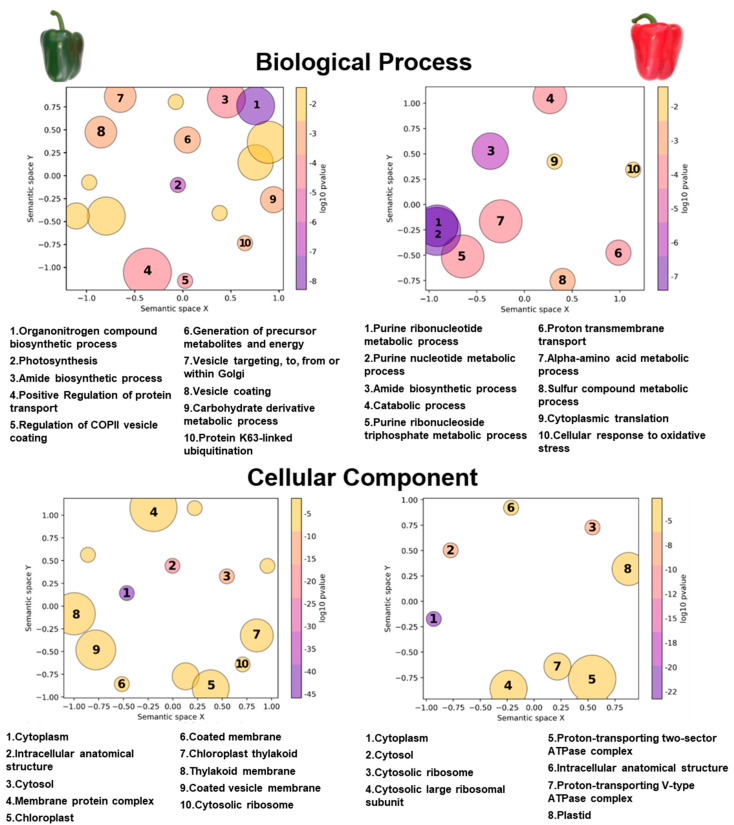
Gene ontology (GO) functional classification obtained for persulfidated proteins of pepper fruits. The scatterplots show GO terms as circles arranged such that those that are most similar in semantic space X and Y are placed nearest to each other. In this case, those proteins that appear exclusively at each stage of ripening have been analyzed, either green fruits (left panels) or red fruits (right panels), and the most enriched terms are shown. The number that appears inside each circle indicates which category proteins belong to. The size of the circles is directly proportional to the number of proteins in each sub-category that fall under each main category according to our data. The distance between the circles indicates a direct relationship between the categories. The *p*-value indicates the statistical significance of the GO category enrichment in our results.

To elucidate the potential functions of persulfidation during pepper fruit ripening, we performed a functional enrichment analysis based on Gene Ontology (GO) terms (*p* < 0.05) (Figure 2). In our analysis of the persulfidated proteins uniquely identified in green and red bell pepper fruits, distinct patterns emerged regarding their involvement in various biological processes. For green pepper fruits, the most enriched terms corresponded to essential processes such as the biosynthesis of nitrogen-containing organic compounds, photosynthesis, as well as protein transport and vesicle trafficking between the endoplasmic reticulum and the Golgi apparatus. Furthermore, the analysis revealed the predominant distribution of these proteins within the cytoplasm and chloroplasts. Conversely, in red pepper fruits, persulfidated proteins exhibited a different spectrum of biological functions. The protein groups with higher enriched values were primarily associated with the metabolism of purine-based molecules, encompassing ribonucleotides and deoxyribonucleotides, the biosynthesis of amide compounds, amino acids, and proteins, as well as participation in the cellular response to oxidative stress. This indicates a distinct metabolic and regulatory landscape in red pepper fruits, underscoring the diverse roles of persulfidation in modulating cellular processes at different stages of fruit development and ripening.

**Figure 3:**
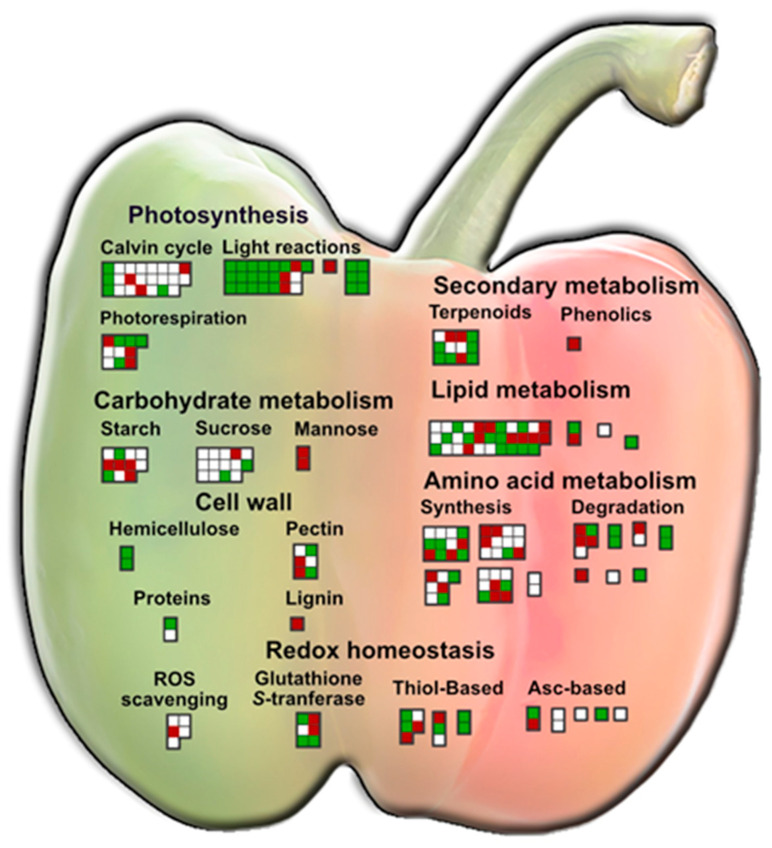
MapMan categories assigned to the sweet pepper fruit persulfidome. Qualitative analysis of the diverse physiological aspects in which the proteins identified as potential persulfidation targets are involved. Each box represents a protein, color-coded to denote its presence in specific ripening stages of the fruit. Proteins exclusively identified in immature fruits are depicted in green, those exclusive to ripe fruits are shown in red, and proteins shared in both ripening stages are depicted in white.

To provide a more comprehensive physiological context, we categorized the identified proteins using the Mapman nomenclature. This classification revealed that the persulfidated proteins span 29 categories, each covering various plant metabolic aspects. Notably, over 26% of the proteins (246) were associated with protein metabolism, encompassing processes such as biosynthesis (89), homeostasis (114), modifications (28), and translocations (18). Additionally, significant representation was observed in primary metabolic pathways, including photosynthesis (74), amino acid (56), carbohydrate (50), and lipid (41) metabolism. While exhibiting fewer proteins, categories such as redox homeostasis (35) and secondary metabolism (14) were noteworthy, constituting 3.7% and 1.8% of the persulfidated proteins, respectively. Figure 3 provides a schematic summary of these findings. As shown in the figure, the majority of the persulfidated proteins in immature green fruits are especially, as expected, grouped in the photosynthetic pathways. On the contrary, in red fruits, the number of proteins that are related to carbohydrate metabolism is higher, possibly explaining the higher sweetness of these fruits compared to the green ones. Also, the catabolism of proteins seems to be enhanced in ripe fruits as a consequence of degenerative processes that are associated with the latest stages of fruit development. Supplementary Table S4 displays the list of the proteins shown in the MapMan categories of Figure 3.

Notwithstanding the persulfidome provides us with a list of proteins that are susceptible to be persulfidated, it is important to evaluate the effect of this PTM on the function of the identified protein. Thus, as part of the characterization of the persulfidome and to corroborate the effect of the persulfidation events in pepper fruit metabolism, two enzymes were selected: glutathione reductase (GR), an enzyme of the ascorbate–glutathione cycle that depends on NADPH to regenerate reduced glutathione (GSH), and leucine aminopeptidase (LAP), an enzyme that has recently been identified in pepper fruits to be modulated by nitric oxide (NO), another signal molecule that mediates different PTMs such as nitration and S-nitrosation [31]. Both enzymes were detected in green and red fruits and were assigned to chloroplasts, according to their deduced amino acid sequences. This analysis of the redox and the peptide/amino acid metabolisms from a persulfidation perspective may shed light on a potential link between both processes, not explored thus far, thus contributing to envisage glutathione as a relevant metabolite in the physiology of pepper fruits. In fact, as well as being involved in the antioxidant metabolism, glutathione could be persulfidated, but it could also be broken down by LAP activity.

**Figure 4:**
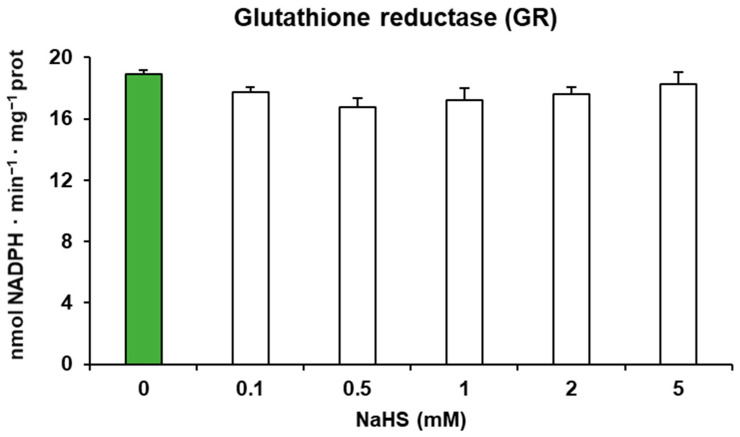
Effect of NaHS on pepper fruit glutathione reductase (GR) activity. Green pepper extracts were pre-treated in the absence or presence of 0.1–5.0 mM NaHS for 1 h at 25 °C (white bars). The green bar corresponds to the control sample not treated with NaHS. Data are shown as the means ± SEM. No statistically significant differences were observed at *p* < 0.05 in relation to the control value.

Figure 4 shows that GR activity preincubated with different concentrations of NaHS, as an H_2_S donor, had no effect at any of the concentrations tested.

**Figure 5:**
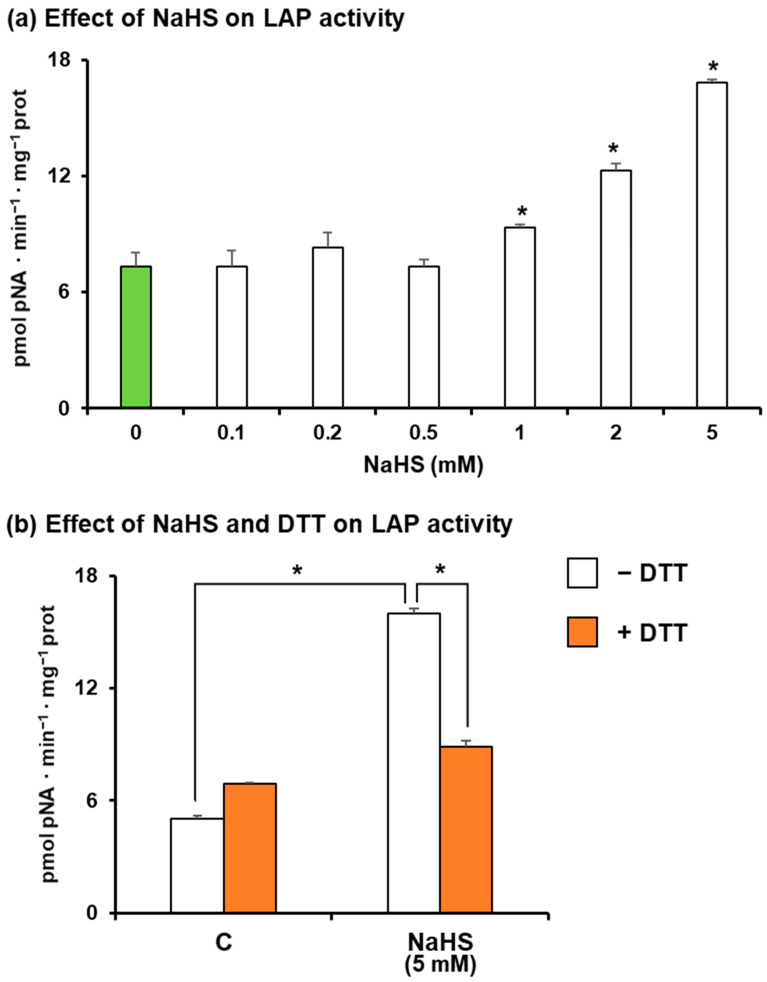
(**a**) Effect of NaHS on the LAP activity of sweet pepper fruits where the samples were pre-treated in the absence or presence of 0.1–5.0 mM NaHS for 1 h at 25 °C (white bars). The green bar corresponds to the control sample not treated with NaHS (**b**) Effect of 5 mM NaHS on LAP activity in the presence and absence of dithiothreitol (DTT). Green pepper extracts were pre-treated in the absence or presence of 5 mM NaHS for 1 h at 25 °C (white bars), and then further incubation with 5 mM DTT was performed for 30 min (brown bars). Data are shown as the means ± SEM. Asterisks indicate that differences in relation to corresponding control values were statistically significant at *p* < 0.05.

On the other hand, Figure 5a displays the in vitro analysis of pepper LAP activity in similar conditions to those used in Figure 4. In this case, it is observed that the pretreatment of the pepper extracts with 1 mM NaHS provoked a slight increase in LAP activity, but this effect increased at higher concentrations (white bars), with LAP activity being 2.5 times higher with 5 mM NaHS. Considering that persulfidation is suggested to be a reversible process, control (C)- and NaHS-treated pepper samples were further incubated with dithiothreitol (DTT). Thus, in Figure 5b, it is observed that the treatment of the control fruit samples with DTT induced a slight increase in the enzyme activity. However, in the samples pretreated with NaHS, the subsequent treatment with DTT reversed its effect by around 50% (brown bars). This indicates that the persulfidation of LAP in sweet pepper fruits is a reversible process which seems to be driven by reducing compounds.

**Figure 6:**
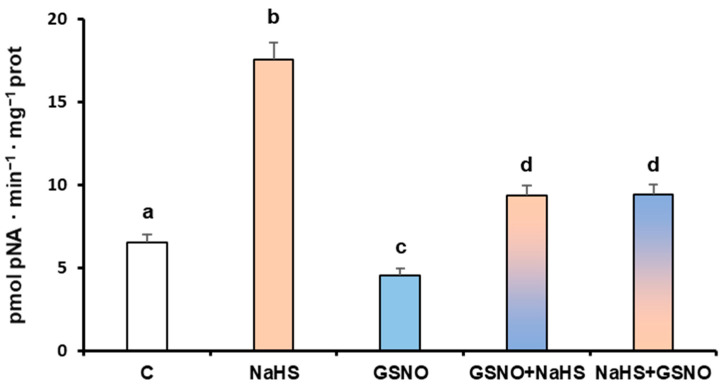
Effect of NaHS and *S*-nitrosoglutathione (GSNO) on the LAP activity of sweet pepper fruit. Green pepper extracts were only treated with either 5 mM NaHS or 5 mM GSNO for 1 h at 25 °C. Additionally, samples were treated with the two compounds: first adding GSNO for 30 min at 25 °C and further incubating with NaHS for another 30 min at the same temperature (column GSNO + NaHS), and vice versa (column NaHS + GSNO). The white bar corresponds to the control sample without any treatment. The light brown bar corresponds to the treatment with NaHS. The blue bar corresponds to the GSNO treatment. The bars with both colors correspond to a combined and sequenced treatment, with the lower color, either blue (GSNO) or light brown, being applied first. Data are shown as the means ± SEM. Different letters indicate significant differences (*p* < 0.05).

In a previous study using *S*-nitrosoglutathione (GSNO) and nitroso-cysteine (NO-Cys), it was found that these NO donors caused the downregulation of the pepper LAP activity. However, this inhibition was not due to the NO released from these compounds because when GSH and Cys were used as internal controls, the same downregulation of LAP activity was also observed, thus indicating that these effects were due to the reductants [31]. To get deeper insights into the modulation of LAP activity, the pepper fruit samples were incubated with GSNO and NaHS that may compete for the same Cys residue as a target. Figure 6 shows that when samples were subjected to a dual incubation with NaHS and GSNO, regardless of whether the pepper samples were first incubated with NaHS or with GSNO, the final effect was an inhibition. This suggests that the effect of the reducing compounds prevails over the positive effect of the H_2_S.

## 4. Discussion

Hydrogen sulfide (H_2_S) has gained great relevance in plant metabolism since it exerts signaling functions in numerous physiological processes [46,47,48,49,50] and in response to biotic and abiotic stresses [8,33,51,52]. In fact, H_2_S is exogenously applied to vegetables and fruits using donors such as NaHS since it exerts positive effects. For example, in spinach, H_2_S increases resistance to drought [53]; in lettuce, it increases shoot elongation [54]; in ginger, it alleviates the toxic effects of cadmium by increasing the activity of different antioxidant enzymes [55]; in banana, it reduces chilling injury during postharvest [56,57,58]; in strawberry, H_2_S prolongs the shelf-life and reduces the decay rate of harvested fruits [59,60]; in cold-stored peaches, H_2_S regards fruit softening and avoid flesh browning [61,62]; and in apples, it promotes resistance against *Penicillium expansum* [63].

One of the mechanisms by which H_2_S regulates protein functions is by an oxiPTM named persulfidation [64,65,66], and the bulk of the proteins that undergo persulfidation are designated as the persulfidome. Research on the persulfidome is more advanced in animal cells [67,68,69,70,71,72], while in plants, as mentioned previously, most studies have focused on Arabidopsis, and to our knowledge, the persulfidome of any fruit is still unknown, as are the ways in which it could be modulated by ripening events since most studies have focused thus far on the Arabidopsis model plant under physiological and stress conditions [8,17,32,73,74,75]. Although the Arabidopsis persulfidome has been investigated mainly in leaves [17], whose metabolism is far different from that of the pepper fruits, the comparative analysis indicated that only 402 (25%) of the total proteins identified were shared by both organs and species. Furthermore, from the total persulfidated protein detected in pepper fruits, it is remarkable that 394 and 248 proteins were exclusively present in green and red pepper fruits, respectively. These data reflect the great metabolic difference between both stages of ripening, a physiological process that is characterized by an active nitro-oxidative metabolism [28].

Currently, among the potential persulfidated proteins identified in plant cells, few have been investigated to evaluate how this PTM could affect their function. Exogenous applications of H_2_S have been reported to alter some proteins whose activity was upregulated, such as RuBisCO and O-acetylserine(thiol)lyase [76], L-cysteine desulfhydrase (LCD) [77], ascorbate peroxidase (APX) [32,78], glyceraldehyde 3-phosphate dehydrogenase (GAPDH) [32], respiratory burst oxidase homolog protein D (RBOHD) [79] and L-cysteine desulfhydrase DES1 [77], transcription factor ABSCISIC ACID INSENSITIVE 4 (ABI4) [80], mitogen-activated protein kinase (MPK) 4 [81], peroxidase (Li et al. [78]), SNF1-RELATED PROTEIN KINASE2.6 (SnRK2.6) [77,82], and nitrate reductase [83].

On the other hand, there is a group of enzymes whose activity has been observed to be downregulated by H_2_S, including glutamine synthetase (GS) [32] and 1-aminocyclopropane-1-carboxylic acid oxidase (ACO) in tomato [84], cysteine protease ATG4 [85], ATG18a [33], NADP-isocitrate dehydrogenase (NADP-ICDH) [86], NADP-malic enzyme (NADP-ME) [87], glucose-6-phosphate dehydrogenase (G6PDH) [29], catalase [88], flowering locus C protein (FLC1 and 3) [89], peroxidase IV [27], and lipoxygenase 1 (LOX 1) [90]. More recently, it has been shown that the persulfidation of cytosolic glucose 6-phosphate dehydrogenase in Arabidopsis and tomato can alter their homotetrameric structure and consequently the stability of the enzyme [91]. On the other hand, in Arabidopsis, it has been described that persulfidation mediates the interaction between the plasma membrane H^+^-ATPase (PMA1) and GENERAL REGULATORY FACTOR 4, allowing an increased tolerance to salinity since it enhances the H^+^ efflux and maintains K^+^/Na^+^ homeostasis under salt stress [92].

At present, the most plausible effect of H_2_S at the protein level is the promotion of persulfidation, but this event has been little investigated in fruits and other vegetables. Thus, exogenous H_2_S delayed the ripening process of tomato by persulfidation linked to several proteins, including WRKY6, WRKY71 transcription factor, and E3 ligase BRG3 [93,94], and promoted Arabidopsis flowering by the persulfidation of transcript factor AtU2AF65a [50]; in pear, the persulfidation of transcription factor MYB10 triggered a lower content of anthocyanin on the skin [95]. However, to our knowledge, there is no information about any persulfidome analysis of fleshy fruits during their ripening process. Based on the early information obtained from the Arabidopsis persulfidome [17,35], in previous studies, we selected different pepper fruit proteins involved in the metabolism of reactive oxygen species (ROS) such as catalase, APX, peroxidase, and NADPH-generating enzymes, among others, to evaluate, using in vitro approaches, how they could be regulated by H_2_S, either positively or negatively [28,86]. However, concluding data about the potential persulfidation of those proteins could not be drawn from those analyses. Therefore, the information reported here for pepper fruits provides a set of potential protein targets for persulfidation, in competition with other thiol-based oxiPTMs such as *S*-nitrosation, *S*-glutathionylation, and *S*-sulfenylation, among others, for susceptible cysteines [86,87,96]. Furthermore, according to the experimental design, the common persulfidome from both green and red fruits would allow us to attribute certain roles to specific persulfidated proteins in the ripening process.

In the present study, among the pepper proteins that are endogenously persulfidated, we add new enzymes, such as glutathione reductase (GR), whose activity was unaffected by persulfidation, suggesting that the effect of persulfidation on GR could be linked to other aspects not related to its activity, such as protection against protein overoxidation. On the contrary, leucine aminopeptidase activity was upregulated by this oxiPTM, and this effect was reversed by the reducing agent dithiothreitol (DTT).

GR is a key enzyme in the ascorbate–glutathione cycle that uses NADPH to keep the cellular levels of reduced glutathione (GSH), a non-enzymatic antioxidant that participates in the homeostasis of the cellular redox state [97,98] and which can interact with NO through an *S*-nitrosation event to generate GSNO, a compound that is considered a reservoir of NO in the cell [99,100]. Remarkably, a previous study carried out in pea leaves demonstrated that GR can undergo tyrosine nitration and *S*-nitrosation, but none of these NO-derived PTMs had an effect on its activity, thus suggesting that it could be a mechanism to preserve the cellular GSH content. Therefore, the fact that persulfidation does not affect GR activity would be in concordance with being a mechanism to preserve GSH levels in pepper fruits, whose content decreases during ripening [26].

Plant leucine aminopeptidase (LAP) is a metalloenzyme, often requiring metal ions such as zinc or manganese for its catalytic activity [101,102,103,104]. LAP is typically found in the cytoplasm and plastids but can also be localized in other cellular compartments including mitochondria and peroxisomes, depending on the plant species and specific physiological conditions [31,43,105,106,107,108,109,110]. It plays a significant role in plant development [111,112,113] and its response to environmental adverse conditions including abiotic (Gu et al. [114]; Gu et al. [115]) and biotic stresses [116,117]. LAP typically exhibits broad substrate specificity but has a preference for peptides with a leucine residue at the N-terminus, and is involved in the degradation of peptides into amino acids, particularly by removing leucine residues from the amino terminus of polypeptides. This process is crucial for protein turnover and the recycling of amino acids within plant cells.

Recently, in pepper fruit, we have shown that LAP activity increased during ripening. The enzyme was also a target of NO-derived PTMs (tyrosine nitration and *S*-nitrosation), and *S*-cyanylation, with its activity being either negatively or positively regulated, respectively [31]. This seems to be correlated with the protein degradation associated with the disassembly of some organelles, such as it occurs in chloroplasts that transformed into chromoplasts during pepper ripening. Furthermore, LAP has been suggested to function in the free amino acid regulation, and peptide and protein turnover. Thus, LAP activity has been correlated with GSH recycling during the ripening of durian (*Durio zibethinus* L.) fruits [118]. This fruit is rich in different sulfur-containing compounds including the dipeptide γ-glutamylcysteine and the tripeptide glutathione (γ-glutamyl-cysteinyl-glycine) [119,120], and LAP was shown to have Cys-Gly dipeptidase activity in the γ-glutamyl cycle, which would allow GSH recycling in the fruit. In this sense, Cys is the precursor of sulfur-containing volatiles during durian ripening due to the catabolism of GSH. Likewise, it has been shown that the GSH pool can be mobilized by physiological thiols like Cys and Cys-Gly, which, under certain oxidative conditions and in the presence of certain metal ions, can promote the formation of ROS [121,122,123]. This situation may occur in pepper fruits, thus contributing to the nitro-oxidative metabolism that they undergo during ripening [26], where the GSH could regenerate Cys and Cys-Gly through trans-thiolation reactions. Accordingly, taking into account this last view plus, the role of GSH in the ascorbate–glutathione cycle and the detoxification processes through glutathione-S-transferase (GST), an enzymatic system which is also influenced by the ripening process (Figure 3), GSH could be envisaged as a relevant player in the physiology of pepper fruit. Recently, reactive polysulfides have been detected and quantified in different plant species, including pepper fruits [15]. These polysulfides include cellular low-molecular thiols to form, for example, glutathione persulfide (GSSH), which could be involved in a trans-persulfidation process of protein Cys residues. This process could provide an additional regulation mechanism equivalent to that attributed to *S*-nitrosoglutathione (GSNO), which mediates trans-nitrosation processes [124,125]. However, these mechanisms will need future experimental analyses, although one of the main challenges is the technological difficulty in determining these compounds in plant tissue.

## 5. Conclusions

**Figure 7:**
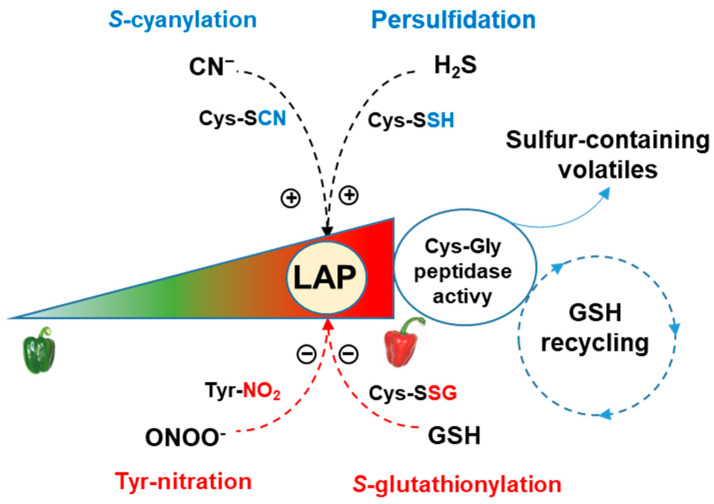
Simple working model of the potential function of the LAP from pepper fruits in the mechanism of the GSH recycling process through its Cys–Gly peptidase activity and generation of sulfur-containing volatiles. During pepper fruit ripening, LAP activity is positively regulated by H_2_S (persulfidation) and cyanide (*S*-cyanylation), but it is negatively regulated by tyrosine nitration and *S*-glutathionylation. Based on our previous model [31].

To the best of our knowledge, the present study provides the first report of the persulfidome of pepper fruit at different ripening stages and provides a new tool to study the relevance of H_2_S metabolism in this process that can be extrapolated as a starting point to other types of fleshy fruits, both climacteric and non-climacteric. Furthermore, it was found that whereas GR activity, which is involved in the regeneration of GSH, was unaffected by H_2_S, LAP activity was upregulated. Based on previous studies where it has been reported that (i) LAP participates in the GSH recycling and the generation of sulfur-containing volatiles involved in the process of fruit ripening [126,127]; (ii) pepper fruits have sulfur-containing volatiles which contribute to their flavor [119,126]; (iii) LAP activity increases during pepper ripening but is downregulated by reductants such as GSH and Cys [31]; and (iv) there is a decline in GSH content during pepper ripening [26], it could be hypothesized that H_2_S is a new element in this network which regulates the GSH content and sulfur-containing volatiles through the upregulation of the LAP. Figure 7 provides a working model that summarizes these new elements and previous ones.

## Data Availability

Data are contained within the article and Supplementary Materials.

## Supplementary Materials

The following supporting information can be downloaded at: https://www.mdpi.com/article/10.3390/antiox13060719/s1, Table S1: The list of all the proteins identified to be endogenously persulfidated in immature green and ripe red pepper fruits; Table S2. Raw data obtained using mass spectrometry analysis; Table S3. The list of persulfidated proteins shared in pepper fruits and Arabidopsis leaves; Table S4. The list of the proteins shown in the MapMan categories.

## References

[1] Abe K., Kimura H. (1996). The possible role of hydrogen sulfide as an endogenous neuromodulator. J. Neurosci..

[2] Kimura H. (2019). Signaling by hydrogen sulfide (H_2_S) and polysulfides (H_2_Sn) in the central nervous system. Neurochem. Int..

[3] Szabo C. (2022). Novel Regulatory Roles of Hydrogen Sulfide in Health and Disease. Biomolecules.

[4] Yamasaki H., Cohen M.F. (2016). Biological consilience of hydrogen sulfide and nitric oxide in plants: Gases of primordial earth linking plant, microbial and animal physiologies. Nitric Oxide.

[5] Iciek M.B., Bilska-Wilkosz A., Kozdrowicki M., Górny M. (2023). Reactive Sulfur Species in Human Diseases. Antioxid. Redox Signal..

[6] Zhang J., Zhou M., Zhou H., Zhao D., Gotor C., Romero L.C., Shen J., Ge Z., Zhang Z., Shen W. (2021). Hydrogen sulfide, a signaling molecule in plant stress responses. J. Integr. Plant Biol..

[7] Khan M.S.S., Islam F., Ye Y., Ashline M., Wang D., Zhao B., Fu Z.Q., Chen J. (2022). The Interplay between Hydrogen Sulfide and Phytohormone Signaling Pathways under Challenging Environments. Int. J. Mol. Sci..

[8] Jurado-Flores A., Aroca A., Romero L.C., Gotor C. (2023). Sulfide promotes tolerance to drought through protein persulfidation in Ar-abidopsis. J. Exp. Bot..

[9] Huang J., Xie Y. (2023). Hydrogen Sulfide Signaling in Plants. Antioxid. Redox Signal..

[10] Filipovic M.R., Zivanovic J., Alvarez B., Banerjee R. (2018). Chemical biology of H_2_S signaling through persulfidation. Chem. Rev..

[11] Cuevasanta E., Lange M., Bonanata J., Coitiño E.L., Ferrer-Sueta G., Filipovic M.R., Alvarez B. (2015). Reaction of Hydrogen Sulfide with Disulfide and Sulfenic Acid to Form the Strongly Nucleophilic Persulfide. J. Biol. Chem..

[12] Sawa T., Motohashi H., Ihara H., Akaike T. (2020). Enzymatic Regulation and Biological Functions of Reactive Cysteine Persulfides and Polysulfides. Biomolecules.

[13] Ogata S., Matsunaga T., Jung M., Barayeu U., Morita M., Akaike T. (2023). Persulfide Biosynthesis Conserved Evolutionarily in All Or-ganisms. Antioxid. Redox Signal..

[14] Abidin Q.H.Z., Ida T., Morita M., Matsunaga T., Nishimura A., Jung M., Hassan N., Takata T., Ishii I., Kruger W. (2023). Synthesis of Sulfides and Persulfides Is Not Impeded by Disruption of Three Canonical Enzymes in Sulfur Metabolism. Antioxidants.

[15] Kasamatsu S., Kinno A., Hishiyama J.-I., Akaike T., Ihara H. (2023). Development of methods for quantitative determination of the total and reactive polysulfides: Reactive polysulfide profiling in vegetables. Food Chem..

[16] Benchoam D., Semelak J.A., Cuevasanta E., Mastrogiovanni M., Grassano J.S., Ferrer-Sueta G., Zeida A., Trujillo M., Möller M.N., Estrin D.A. (2020). Acidity and nucleophilic reactivity of glutathione persulfide. J. Biol. Chem..

[17] Aroca A., Benito J.M., Gotor C., Romero L.C. (2017). Persulfidation proteome reveals the regulation of protein function by hydrogen sulfide in diverse biological processes in Arabidopsis. J. Exp. Bot..

[18] Corpas F.J., González-Gordo S., Muñoz-Vargas M.A., Rodríguez-Ruiz M., Palma J.M. (2021). The *Modus operandi* of hydrogen sulfide(H_2_S)-dependent protein persulfidation in higher plants. Antioxidants.

[19] Tripodi P., Kumar S., Ramchiary N., Kole C. (2019). The Capsicum Crop: An Introduction. The Capsicum Genome. Compendium of Plant Genomes.

[20] Baenas N., Belovi’c N., Ilicb N., Moreno D.A., García-Viguera C. (2019). Industrial use of pepper (*Capsicum annum* L.) derived products: Technological benefits and biological advantages. Food Chem..

[21] Materska M., Perucka I. (2005). Antioxidant activity of the main phenolic compounds isolated from hot pepper fruit (*Capsicum annuum* L.). J. Agric. Food Chem..

[22] Howard L.R., Wildman R.E.C., Wildman R.E.C. (2007). Antioxidant vitamin and phytochemical content of fresh and processed pepper fruit (*Capsicum annuum*). Handbook of Nutraceuticals and Functional Foods.

[23] Wahyuni Y., Ballester A.-R., Sudarmonowati E., Bino R.J., Bovy A.G. (2013). Secondary metabolites of *Capsicum* species and their importance in the human diet. J. Nat. Prod..

[24] Hamed M., Kalita D., Bartolo M.E., Jayanty S.S. (2019). Capsaicinoids, Polyphenols and Antioxidant Activities of *Capsicum annuum*: Comparative Study of the Effect of Ripening Stage and Cooking Methods. Antioxidants.

[25] Zhang L., Zhang F., Wang Y., Ma X., Shen Y., Wang X., Yang H., Zhang W., Lakshmanan P., Hu Y. (2023). Physiological and metabolomic analysis reveals maturity stage-dependent nitrogen regulation of vitamin C content in pepper fruit. Front. Plant Sci..

[26] González-Gordo S., Bautista R., Claros M.G., Cañas A., Palma J.M., Corpas F.J. (2019). Nitric oxide-dependent regulation of sweet pepper fruit ripening. J. Exp. Bot..

[27] González-Gordo S., Muñoz-Vargas M.A., Palma J.M., Corpas F.J. (2023). Class III peroxidases (POD) in pepper (*Capsicum annuum* L.): Genome-wide identification and regulation during nitric oxide (NO)-influenced fruit ripening. Antioxidants.

[28] González-Gordo S., Rodríguez-Ruiz M., López-Jaramillo J., Muñoz-Vargas M.A., Palma J.M., Corpas F.J. (2022). Nitric Oxide (NO) differ-entially modulates the ascorbate peroxidase (APX) isozymes of sweet pepper (*Capsicum annuum* L.) fruits. Antioxidants.

[29] Muñoz-Vargas M.A., González-Gordo S., Taboada J., Palma J.M., Corpas F.J. (2023). In silico RNAseq and biochemical analyses of glucose-6-phosphate dehydrogenase (G6PDH) from sweet pepper fruits: Involvement of nitric oxide (NO) in ripening and modulation. Plants.

[30] Muñoz-Vargas M.A., López-Jaramillo J., González-Gordo S., Paradela A., Palma J.M., Corpas F.J. (2023). H_2_S-generating cytosolic L-cysteine desulfhydrase and mitochondrial D-cysteine desulfhydrase from sweet pepper (*Capsicum annuum* L.) are regulated during fruit ripening and by nitric oxide. Antioxid. Redox Signal..

[31] Muñoz-Vargas M.A., Taboada J., González-Gordo S., Palma J.M., Corpas F.J. (2024). Characterization of leucine aminopeptidase (LAP) activity in sweet pepper fruits during ripening and its inhibition by nitration and reducing events. Plant Cell Rep..

[32] Aroca Á., Serna A., Gotor C., Romero L.C. (2015). S-sulfhydration: A cysteine posttranslational modification in plant systems. Plant Physiol..

[33] Aroca A., Yruela I., Gotor C., Bassham D.C. (2021). Persulfidation of ATG18a regulates autophagy under ER stress in *Arabidopsis*. Proc. Natl. Acad. Sci. USA.

[34] Matamoros M.A., Romero L.C., Tian T., Román Á., Duanmu D., Becana M. (2023). Persulfidation of plant and bacteroid proteins is involved in legume nodule development and senescence. J. Exp. Bot..

[35] Aroca A., Jurado-Flores A., Filipovic M.R., Gotor C., Romero L.C. (2022). Detection of protein persulfidation in plants by the dimedone switch method. Methods Enzymol..

[36] Kolberg L., Raudvere U., Kuzmin I., Adler P., Vilo J., Peterson H. (2023). g:Profiler—Interoperable web service for functional enrichment analysis and gene identifier mapping (2023 update). Nucleic Acids Res..

[37] Reijnders M.J.M.F., Waterhouse R.M. (2021). Summary Visualizations of Gene Ontology Terms With GO-Figure!. Front. Bioinform..

[38] Schwacke R., Ponce-Soto G.Y., Krause K., Bolger A.M., Arsova B., Hallab A., Gruden K., Stitt M., Bolger M.E., Usadel B. (2019). MapMan4: A Refined Protein Classification and Annotation Framework Applicable to Multi-Omics Data Analysis. Mol. Plant.

[39] Bolger M., Schwacke R., Usadel B. (2021). MapMan Visualization of RNA-Seq Data Using Mercator4 Functional Annotations. Methods Mol. Biol..

[40] Thimm O., Bläsing O., Gibon Y., Nagel A., Meyer S., Krüger P., Selbig J., Müller L.A., Rhee S.Y., Stitt M. (2004). MAPMAN: A user-driven tool to display genomics data sets onto diagrams of metabolic pathways and other biological processes. Plant J..

[41] Usadel B., Poree F., Nagel A., Lohse M., Czedik-Eysenberg A., Stitt M. (2009). A guide to using MapMan to visualize and compare Omics data in plants: A case study in the crop species, Maize. Plant Cell Environ..

[42] Camacho C., Coulouris G., Avagyan V., Ma N., Papadopoulos J., Bealer K., Madden T.L. (2009). BLAST+: Architecture and applications. BMC Bioinform..

[43] Corpas F.J., Palma J.M., Del Río L.A. (1993). Evidence for the presence of proteolytic activity in peroxisomes. Eur. J. Cell Biol..

[44] Tuppy H., Wiesbauer U., Wintersberger E. (1962). Amino acid-p-nitroanilide as a substrate for aminopeptidases and other proteolytic enzymes. Hoppe Seylers Z Physiol. Chem..

[45] Edwards E.A., Rawsthorne S., Mullineaux P.M. (1990). Subcellular distribution of multiple forms of glutathione reductase in leaves of pea (*Pisum sativum* L.). Planta.

[46] Fang T., Cao Z., Li J., Shen W., Huang L. (2014). Auxin-induced hydrogen sulfide generation is involved in lateral root formation in tomato. Plant Physiol. Biochem..

[47] Fang H., Liu R., Yu Z., Shao Y., Wu G., Pei Y. (2022). Gasotransmitter H_2_S accelerates seed germination via activating AOX mediated cyanide-resistant respiration pathway. Plant Physiol. Biochem..

[48] Liu Q., Zhou Y., Li H., Liu R., Wang W., Wu W., Yang N., Wang S. (2020). Osmotic stress-triggered stomatal closure requires Phospholipase Dδ and hydrogen sulfide in Arabidopsis thaliana. Biochem. Biophys. Res. Commun..

[49] Dai J., Wen D., Li H., Yang J., Rao X., Yang Y., Yang J., Yang C., Yu J. (2024). Effect of hydrogen sulfide (H_2_S) on the growth and development of tobacco seedlings in absence of stress. BMC Plant Biol..

[50] Ma T., Xu S., Wang Y., Zhang L., Liu Z., Liu D., Jin Z., Pei Y. (2024). Exogenous hydrogen sulphide promotes plant flowering through the *Arabidopsis* splicing factor AtU2AF65a. Plant Cell Environ..

[51] Younis A.A., Mansour M.M.F. (2023). Hydrogen sulfide priming enhanced salinity tolerance in sunflower by modulating ion hemostasis, cellular redox balance, and gene expression. BMC Plant Biol..

[52] Wang J., Dou J., Yue Z., Wang J., Chen T., Li J., Dai H., Dou T., Yu J., Liu Z. (2024). Effect of hydrogen sulfide on cabbage photosynthesis under black rot stress. Plant Physiol. Biochem..

[53] Chen J., Shang Y.-T., Wang W.-H., Chen X.-Y., He E.-M., Zheng H.-L., Shangguan Z. (2016). Hydrogen sulfide-mediated polyamines and sugar changes are involved in hydrogen sulfide-induced drought tolerance in spinacia oleracea seedlings. Front. Plant Sci..

[54] Liu R., Lal R. (2015). Effects of low-level aqueous hydrogen sulfide and other sulfur species on lettuce (*Lactuca sativa*) seed germination. Commun. Soil Sci. Plant Anal..

[55] Chen Z., Liu C., Cao B., Xu K. (2022). A hydrogen sulfide application can alleviate the toxic effects of cadmium on ginger (*Zingiber officinale* Roscoe). Environ. Sci. Pollut. Res..

[56] Ali S., Nawaz A., Naz S., Ejaz S., Maqbool M., Siddiqui M.H., Kalaji H.M., Wróbel J., Telesiński A., Auriga A. (2022). Hydrogen sulfide mitigates chilling injury of postharvest banana fruits by regulating γ-aminobutyric acid shunt pathway and ascorbate–glutathione cycle. Front. Plant Sci..

[57] Li D., Limwachiranon J., Li L., Du R., Luo Z. (2016). Involvement of energy metabolism to chilling tolerance induced by hydrogen sulfide in cold-stored banana fruit. Food Chem..

[58] Ge Y., Hu K.D., Wang S.S., Hu L.Y., Chen X.Y., Li Y.H., Yang Y., Yang F., Zhang H. (2017). Hydrogen sulfide alleviates postharvest ripening and senescence of banana by antagonizing the effect of ethylene. PLoS ONE.

[59] Hu L.-Y., Hu S.-L., Wu J., Li Y.-H., Zheng J.-L., Wei Z.-J., Liu J., Wang H.-L., Liu Y.-S., Zhang H. (2012). Hydrogen sulfide prolongs postharvest shelf life of strawberry and plays an antioxidative role in fruits. J. Agric. Food Chem..

[60] Zhang C., Shi J.Y., Zhu L.P., Li C.L., Wang Q.G. (2014). Cooperative effects of hydrogen sulfide and nitric oxide on delaying softening and decay of strawberry. Int. J. Agric. Biol. Eng..

[61] Yu M., Chen Y., Zhu Q., Wu X., Jiang S., Wei Y., Ye J., Xu F., Shao X. (2024). Hydrogen sulfide mediated methyl jasmonate -induced cold resistance in peach fruit. Postharvest Biol. Technol..

[62] Zhao Y., Zhu D., Zhao L., Luo Y., Li J., Xie B., Liu Y., Bao Y., Wu Z., Zheng Y. (2024). Hydrogen sulfide retards fruit softening and prevents flesh browning in cold-stored peaches by regulating cell wall-modifying enzymes, phenolic, and proline metabolism. Postharvest Biol. Technol..

[63] Deng H., Wang B., Liu Y., Ma L., Zong Y., Prusky D., Bi Y. (2021). Sodium hydrosulfide induces resistance against *Penicillium expansum* in apples by regulating hydrogen peroxide and nitric oxide activation of phenylpropanoid metabolism. Front. Microbiol..

[64] Yang C.-T., Devarie-Baez N.O., Hamsath A., Fu X.-D., Xian M. (2020). S-Persulfidation: Chemistry, Chemical Biology, and Significance in Health and Disease. Antioxid. Redox Signal..

[65] Luo S., Kong C., Ye D., Liu X., Wang Y., Meng G., Han Y., Xie L., Ji Y. (2023). Protein Persulfidation: Recent Progress and Future Directions. Antioxid. Redox Signal..

[66] Vignane T., Filipovic M.R. (2023). Emerging Chemical Biology of Protein Persulfidation. Antioxid. Redox Signal..

[67] Gao X.H., Krokowski D., Guan B.J., Bederman I., Majumder M., Parisien M., Diatchenko L., Kabil O., Willard B., Banerjee R. (2015). Quantitative H_2_S-mediated protein sulfhydration reveals metabolic reprogramming during the integrated stress response. Elife.

[68] Wu Q., Zhao B., Weng Y., Shan Y., Li X., Hu Y., Liang Z., Yuan H., Zhang L., Zhang Y. (2019). Site-Specific Quantification of Persulfidome by Combining an Isotope-Coded Affinity Tag with Strong Cation-Exchange-Based Fractionation. Anal. Chem..

[69] Zivanovic J., Kouroussis E., Kohl J.B., Adhikari B., Bursac B., Schott-Roux S., Petrovic D., Miljkovic J.L., Thomas-Lopez D., Jung Y. (2019). Selective Persulfide Detection Reveals Evolutionarily Conserved Antiaging Effects of S-Sulfhydration. Cell Metab..

[70] Fu L., Liu K., He J., Tian C., Yu X., Yang J. (2020). Direct Proteomic Mapping of Cysteine Persulfidation. Antioxid. Redox Signal..

[71] Bithi N., Link C., Henderson Y.O., Kim S., Yang J., Li L., Wang R., Willard B., Hine C. (2021). Dietary restriction transforms the mammalian protein persulfidome in a tissue-specific and cystathionine γ-lyase-dependent manner. Nat. Commun..

[72] Bai J., Jiao F., Salmeron A.G., Xu S., Xian M., Huang L., Chen D.-B. (2023). Mapping Pregnancy-dependent Sulfhydrome Unfolds Diverse Functions of Protein Sulfhydration in Human Uterine Artery. Endocrinology.

[73] García-Calderón M., Vignane T., Filipovic M.R., Ruiz M.T., Romero L.C., Márquez A.J., Gotor C., Aroca A. (2023). Persulfidation protects from oxidative stress under nonphotorespiratory conditions in Arabidopsis. New Phytol..

[74] Jurado-Flores A., Gotor C., Romero L.C. (2023). Proteome Dynamics of Persulfidation in Leaf Tissue under Light/Dark Conditions and Carbon Deprivation. Antioxidants.

[75] Jurado-Flores A., Romero L.C., Gotor C. (2021). Label-free quantitative proteomic analysis of nitrogen starvation in arabidopsis root reveals new aspects of H_2_S signaling by protein persulfidation. Antioxidants.

[76] Chen J., Wu F.-H., Wang W.-H., Zheng C.-J., Lin G.-H., Dong X.-J., He J.-X., Pei Z.-M., Zheng H.-L. (2011). Hydrogen sulphide enhances photosynthesis through promoting chloroplast biogenesis, photosynthetic enzyme expression, and thiol redox modification in *Spinacia oleracea* seedlings. J. Exp. Bot..

[77] Chen S., Jia H., Wang X., Shi C., Wang X., Ma P., Wang J., Ren M., Li J. (2020). Hydrogen sulfide positively regulates abscisic acid signaling through persulfidation of SnRK2.6 in guard cells. Mol. Plant.

[78] Li J., Shi C., Wang X., Liu C., Ding X., Ma P., Wang X., Jia H. (2020). Hydrogen sulfide regulates the activity of antioxidant enzymes through persulfidation and improves the resistance of tomato seedling to Copper Oxide nanoparticles (CuO NPs)-induced oxidative stress. Plant Physiol. Biochem..

[79] Shen J., Zhang J., Zhou M., Zhou H., Cui B., Gotor C., Romero L.C., Fu L., Yang J., Foyer C.H. (2020). Persulfidation-based modification of cysteine desulfhydrase and the NADPH oxidase RBOHD controls guard cell abscisic acid signaling. Plant Cell.

[80] Zhou M., Zhang J., Shen J., Zhou H., Zhao D., Gotor C., Romero L.C., Fu L., Li Z., Yang J. (2021). Hydrogen sulfide-linked persulfidation of ABI4 controls ABA responses through the transactivation of MAPKKK18 in Arabidopsis. Mol. Plants.

[81] Du X., Jin Z., Liu Z., Liu D., Zhang L., Ma X., Yang G., Liu S., Guo Y., Pei Y. (2021). H_2_S persulfidated and increased kinase activity of MPK4 to response cold stress in Arabidopsis. Front. Mol. Biosci..

[82] Chen S., Wang X., Jia H., Li F., Ma Y., Liesche J., Liao M., Ding X., Liu C., Chen Y. (2021). Persulfidation-induced structural change in SnRK2.6 establishes intramolecular interaction between phosphorylation and persulfidation. Mol. Plant.

[83] Zhou H., Zhou Y., Zhang F., Guan W., Su Y., Yuan X., Xie Y. (2021). Persulfidation of nitrate reductase 2 is involved in L-cysteine desulfhydrase-regulated rice drought tolerance. Int. J. Mol. Sci..

[84] Jia H., Chen S., Liu D., Liesche J., Shi C., Wang J., Ren M., Wang X., Yang J., Shi W. (2018). Ethylene-induced hydrogen sulfide negatively regulates ethylene biosynthesis by persulfidation of ACO in tomato under osmotic stress. Front. Plant Sci..

[85] Laureano-Marín A.M., Aroca A., Perez-Perez M.E., Yruela I., Jurado-Flores A., Moreno I., Crespo J.L., Romero L.C., Gotor C. (2020). Abscisic acid-triggered persulfidation of cysteine protease ATG4 mediates regulation of autophagy by sulfide. Plant Cell.

[86] Muñoz-Vargas M.A., González-Gordo S., Cañas A., López-Jaramillo J., Palma J.M., Corpas F.J. (2018). Endogenous hydrogen sulfide (H_2_S) is up-regulated during sweet pepper (*Capsicum annuum* L.) fruit ripening. In vitro analysis shows that NADP-dependent isocitrate dehydrogenase (ICDH) activity is inhibited by H_2_S and NO. Nitric Oxide.

[87] Muñoz-Vargas M.A., González-Gordo S., Palma J.M., Corpas F.J. (2020). Inhibition of NADP-malic enzyme activity by H_2_S and NO in sweet pepper (*Capsicum annuum* L.) fruits. Physiol. Plant..

[88] Corpas F.J., Barroso J.B., González-Gordo S., Muñoz-Vargas M.A., Palma J.M. (2019). Hydrogen sulfide: A novel component in *Arabidopsis* peroxisomes which triggers catalase inhibition. J. Integr. Plant Biol..

[89] Ma X., Zhang L., Pei Z., Zhang L., Liu Z., Liu D., Hao X., Jin Z., Pei Y. (2021). Hydrogen sulfide promotes flowering in heading Chinese cabbage by S-sulfhydration of BraFLCs. Hortic. Res..

[90] González-Gordo S., López-Jaramillo J., Palma J.M., Corpas F.J. (2023). Soybean (*Glycine max* L.) Lipoxygenase 1 (LOX 1) Is Modulated by Nitric Oxide and Hydrogen Sulfide: An In Vitro Approach. Int. J. Mol. Sci..

[91] Wang X., Shi C., Hu Y., Ma Y., Yi Y., Jia H., Li F., Sun H., Li T., Wang X. (2023). Persulfidation maintains cytosolic G6PDs activity through changing tetrameric structure and competing cysteine sulfur oxidation under salt stress in Arabidopsis and tomato. New Phytol..

[92] Ma Y., Li F., Yi Y., Wang X., Li T., Wang X., Sun H., Li L., Ren M., Han S. (2023). Hydrogen sulfide improves salt tolerance through persulfidation of PMA1 in Arabidopsis. Plant Cell Rep..

[93] Sun C., Yao G.-F., Li L.-X., Li T.-T., Zhao Y.-Q., Hu K.-D., Zhang C., Zhang H. (2023). E3 ligase BRG3 persulfidation delays tomato ripening by reducing ubiquitination of the repressor WRKY71. Plant Physiol..

[94] Zhang M., Hu K., Ma L., Geng M., Zhang C., Yao G., Zhang H. (2024). Persulfidation and phosphor-ylation of transcription factor SlWRKY6 differentially regulate tomato fruit ripening. Plant Physiol..

[95] Yao G., Gou S., Zhong T., Wei S., An X., Sun H., Sun C., Hu K., Zhang H. (2023). Persulfidation of transcription factor MYB10 inhibits an-thocyanin synthesis in red-skinned pear. Plant Physiol..

[96] Corpas F.J., González-Gordo S., Rodríguez-Ruiz M., Muñoz-Vargas M.A., Palma J.M. (2022). Thiol-based oxidative posttranslational modifications (oxiPTMs) of plant proteins. Plant Cell Physiol..

[97] Couto N., Wood J., Barber J. (2016). The role of glutathione reductase and related enzymes on cellular redox homoeostasis network. Free Radic. Biol. Med..

[98] Dorion S., Ouellet J.C., Rivoal J. (2021). Glutathione Metabolism in Plants under Stress: Beyond Reactive Oxygen Species Detoxification. Metabolites.

[99] Broniowska K.A., Diers A.R., Hogg N. (2013). *S*-nitrosoglutathione. Biochim. Biophys. Acta.

[100] Saini D., Bapatla R.B., Vemula C.K., Gahir S., Bharath P., Gupta K.J., Raghavendra A.S. (2023). Moderate modulation by *S*-nitrosoglutathione of photorespiratory enzymes in pea (*Pisum sativum*) leaves, compared to the strong effects of high light. Protoplasma.

[101] Pallavicini C., Peruffo A.D.B., Santoro M. (1981). Isolation and partial characterization of grape aminopeptidase. J. Agric. Food Chem..

[102] Casano L.M., Desimone M., Trippi V.S. (1989). Proteolytic Activity at Alkaline pH in Oat Leaves, Isolation of an Aminopeptidase. Plant Physiol..

[103] Matsui M., Fowler J.H., Walling L.L. (2006). Leucine aminopeptidases: Diversity in structure and function. Biol. Chem..

[104] Oszywa B., Makowski M., Pawełczak M. (2013). Purification and partial characterization of aminopeptidase from barley (*Hordeum vulgare* L.) seeds. Plant Physiol. Biochem..

[105] Herbers K., Willmitzer L., Prat S. (1994). Functional analysis of a leucine aminopeptidase from *Solanum tuberosum* L. Planta.

[106] Kang H., Hahn T., Chung I., Park J. (1999). Characterization of an Aminopeptidase from Grapes. Int. J. Plant Sci..

[107] Desimone M., Krüger M., Wessel T., Wehofsky M., Hoffmann R., Wagner E. (2000). Purification and characterization of an aminopeptidase from the chloroplast stroma of barley leaves by chromatographic and electrophoretic methods. J. Chromatogr. B Biomed. Sci. Appl..

[108] Narváez-Vásquez J., Tu C.-J., Park S.-Y., Walling L.L. (2007). Targeting and localization of wound-inducible leucine aminopeptidase A in tomato leaves. Planta.

[109] Tu C.-J., Park S.-Y., Walling L.L. (2003). Isolation and characterization of the neutral leucine aminopeptidase (*LapN*) of tomato. Plant Physiol..

[110] Kolehmainen L., Mikola J. (1971). Partial purification and enzymatic properties of an aminopeptidase from barley. Arch. Biochem. Biophys..

[111] Gupta V.K., Pawar V.S. (1974). Leucine Aminopeptidase activity in tall and dwarf cultivars of rice at successive stages of development. Ann. Bot..

[112] Murphy A., Peer W.A., Taiz L. (2000). Regulation of auxin transport by aminopeptidases and endogenous flavonoids. Planta.

[113] Mahagamasekera M.G.P., Leung D.W.M. (2001). Development of leucine aminopeptidase activity during daylily flower growth and senescence. Acta Physiol. Plant..

[114] Gu Y.-Q., Chao W.S., Walling L.L. (1996). Localization and post-translational processing of the wound-induced leucine aminopeptidase proteins of tomato. J. Biol. Chem..

[115] Gu Y.Q., Walling L.L. (2000). Specificity of the wound-induced leucine aminopeptidase LAP-A) of tomato: Activity on dipeptide and tripeptide substrates. Eur. J. Biochem..

[116] Scranton M.A., Yee A., Park S.-Y., Walling L.L. (2012). Plant leucine aminopeptidases moonlight as molecular chaperones to alleviate stress-induced damage. J. Biol. Chem..

[117] Pautot V., Holzer F.M., Chaufaux J., Walling L.L. (2001). The induction of tomato leucine aminopeptidase genes LapA after *Pseudomonas syringae* pv. *tomato* infection is primarily a wound response triggered by coronatine. Mol. Plant Microbe Interact..

[118] Panpetch P., Sirikantaramas S. (2021). Fruit ripening-associated leucylaminopeptidase with cysteinylglycine dipeptidase activity from durian suggests its involvement in glutathione recycling. BMC Plant Biol..

[119] Naef R., Velluz A., Jaquier A. (2007). New volatile sulfur-containing constituents in a simultaneous distillation-extraction extract of red bell peppers (*Capsicum annuum*). J. Agric. Food Chem..

[120] Du X., Song M., Rouseff R. (2011). Identification of new strawberry sulfur volatiles and changes during maturation. J. Agric. Food Chem..

[121] Del Corso A., Vilardo P.G., Cappiello M., Cecconi I., Dal Monte M., Barsacchi D., Mura U. (2002). Physiological thiols as promoters of glutathione oxidation and modifying agents in protein S-thiolation. Arch. Biochem. Biophys..

[122] Ito T., Ohkama-Ohtsu N. (2023). Degradation of glutathione and glutathione conjugates in plants. J. Exp. Bot..

[123] Kumar S., Kaur A., Chattopadhyay B., Bachhawat A.K. (2015). Defining the cytosolic pathway of glutathione degradation in *Arabidopsis thaliana*: Role of the ChaC/GCG family of γ-glutamyl cyclotransferases as glutathione-degrading enzymes and AtLAP1 as the Cys-Gly peptidase. Biochem. J..

[124] Guerra D., Ballard K., Truebridge I., Vierling E. (2016). S-Nitrosation of Conserved Cysteines Modulates Activity and Stability of *S*-Nitrosoglutathione Reductase (GSNOR). Biochemistry.

[125] Mata-Pérez C., Padilla M.N., Sánchez-Calvo B., Begara-Morales J.C., Valderrama R., Chaki M., Aranda-Caño L., Moreno-González D., Molina-Díaz A., Barroso J.B. (2020). Endogenous Biosynthesis of *S*-Nitrosoglutathione from Nitro-Fatty Acids in Plants. Front. Plant Sci..

[126] Starkenmann C., Niclass Y. (2011). New cysteine-S-conjugate precursors of volatile sulfur compounds in bell peppers (*Capsicum annuum* L. cultivar). J. Agric. Food Chem..

[127] Cannon R.J., Ho C.-T. (2018). Volatile sulfur compounds in tropical fruits. J. Food Drug Anal..

